# Some Handicaps Which the Country Doctor May Overcome

**Published:** 1912-10

**Authors:** B. G. R. Williams

**Affiliations:** Paris, Ill., Author of “Laboratory Methods. With Special Reference to the Needs of the General Practitioner.” etc., etc.


For Texas Medical Journal.

### Some Handicaps Which the Country Doctor May Overcome.

BY B. G. R. WILLIAMS, M. D., PARIS, ILL.,

Author of "Laboratory Methods. With Special Reference to the Needs of the General Practitioner." etc., etc.

Moliere has told us that it is possible to make compromises with heaven. In medicine we make compromises almost daily. Classical description and orthodox practice must be modified to suit the needs of the man outside the luxuries of medicine.

If a man would learn to pray, let him go often to the sea. It is easy enough to commit to memory text-book descriptions of operative technic; but, out upon the desert where hospital conveniences are lacking, many substitutes must be used by the surgeon. After all, what is really needed is intellect.

During the past few years the medical laboratory has gained an important place in medicine. Now the question arises in the mind of the man in the rural district, "May I here, with my limited training, time and resources, carry out some of the more important of these tests?" The writer would answer this question in the affirmative.

Here is an instance which is, after all, but one of many which have attracted my attention during the past few years in which I have been interested in this subject. A physician who entered practice several years before chemical and microscopic aids were introduced into our medical schools, and located in a neighborhood where he had not the advantage of a large laboratory, referred several cases to the city for diagnosis; cases which had proved too much for ordinary methods unaided. By simple examinations, these diseases were accurately diagnosticated and treated. He followed them rather closely for the next few years. Few of these ever returned to him for treatment, and several even went so far as to criticise his fogyism. He also noted that several persons, whom he did not send to these city men, finally drifted in that direction, nevertheless, and returned to the neighborhood with damaging testimony as to his unfitness. The doctor thought it over and, chancing to see one of my articles along this very line, ventured to write to me. Today this man maintains in one corner of his office a special little table, and upon this a microscope and a few chemicals. This, he proudly tells me, has saved him many dollars and much embarrassment. When lack of time or equipment prevent him from carrying out certain examinations, such he has done in the city. But now he understands the principles of the laboratory aid, and by his progressiveness has taught his patients to respect his opinion.

I trust that what I shall say in this communication will be treasured by the reader as valuable information, for I assure you that it comes from the heart of a practitioner of medicine and not from an ordinary chemist or an ultrascientific faddist. It seems that the general practitioner is about to come into his own. For during the past few months the laboratory idea, which has been slowly gaining ground for many years, has attained a greater strength than many men realize; and it is time that these things be pointed out to the man who is lagging. Already our medical schools are turning out graduates fitted for the more important departments of analytical work, and the man who has not had this advantage is forced to compete with methods which will eventually show him up. Thus far you and your future may have been spared, but great changes may come with the next week.

Here is one of many examples. A certain man passed large quantities of blood in his urine. There was considerable pain of an abdominal nature. Attacks of this trouble were treated by the family physician for years by means of irrigations, diuretics, etc. One of these younger men located in that town. Within a week this patient called at his office. The new physician gave him a rather complete examination, in the course of which he carried out some blood tests. Here he ran across a clew, and examination was directed to another point. An appendectomy was finally advised. The patient demurred and sought the counsel of the older. Said the latter:

"Bosh! I have studied your case thoroughly. This young fellow can tell nothing by playing with your blood. For this blood to come from your appendix, it must pass from your intestine, and not from your bladder."

Possibly this man read uncertainty in the older doctor's manner. Anyhow, he risked an operation. In consequence, the older doctor lost within a few weeks much that it had taken many years to gain. Yes, he also began to brighten up on these things; but the younger man had the start, and this proved fatal. Many physicians nowadays intend to get into this work, but through faint heart or the hour's exigencies fail to get busy until one of these emigrants or some fellow in a nearby town puts one over on them.

I am willing to acknowledge that many of those analytical procedures foisted upon the practitioner during the past twenty-five years have proven absolutely worthless. But it is just as well to realize that certain others have stood the test of time and have proven priceless to the man who desires to diagnosticate best and to treat best his patients and to command respect among men.

As Moliere has said, it is possible to make compromises with heaven. There is a feeling among many of the older practitioners that they will never be able to carry out even the more practical diagnostic tests. But, as a matter of fact, we have learned that these really valuable tests may be very easily carried out even by the older man with but little study which he will find absorbing, with but little expense which will prove a good investment for the future and with very little difficulty or loss of time, and he will become absorbed in the work and acknowledge it to be a good investment.

The very future of the man who will not listen is threatened. The laboratory idea, as founded by the great Virchow and Pasteur, occupies no uncertain ground and will not, like the popular song of today, be forgotten tomorrow. Many men have foreseen this future and are beating their colleagues to it. Parenthetically, it is interesting to note that in many cases the older ones are setting the pace.

Doctor, where do you stand? Perhaps you think that you have not had a fair chance—certainly you have not had equal opportunities. But it is not yet too late to get right. I have interested myself in this matter for years, and you may have read other articles written by me. I see men who are doing these things who previously never looked through a microscope nor worked with chemicals. I have carried on a large correspondence with these men, and know whereof I speak. I am always willing to answer questions if I am able to do so, providing return postage is always enclosed. I can only point out the main truths in a paper of this scope, thus hoping to spur the reader to make the start.

Think it over, doctor. It is the "eventually" of the morrow. It is in your journals, your books and your medical society.

Start now and start right. You will meet a few difficulties, but do not despair. Remember that straight, smooth roads do not lead to success, but that the feeble rays of a tiny lantern may illuminate many a stone which would otherwise stub the toe. Do not outfit yourself with impossible and expensive apparatus and equipment or books intended for the research worker and the student, but hold fast only to the truly practical things. Carry out the tests along with your reading if you really wish to learn best. Stick to it and it will soon begin to seem easy. You will grow interested and then enthusiastic. Your neighbor's sneer will be but as the hiss of the goose to whom God gave no sense.

Doctor, do it now. It will pay you. It will save you. Else the difficulties of today will prove the impossibilities of tomorrow.

[A notice of Dr. Williams' book appeared in the July number.—ED.]

